# An Evaluation of the Edible Value of *Salvia miltiorrhiza* Seeds: Proximate Composition, Phytochemical Components and Antioxidant Activity

**DOI:** 10.3390/molecules29071483

**Published:** 2024-03-27

**Authors:** Ruixue Deng, Xueli Ren, Dongjie Liu, Zongyuan Lu, Pu Liu

**Affiliations:** School of Chemistry and Chemical Engineering, Henan University of Science and Technology, Kaiyuan Avenue No. 263, Luoyang 471000, China; dengliu20022002@163.com (R.D.); renxueli1124@163.com (X.R.); d15236162521@163.com (D.L.); lzy@nature-standard.com (Z.L.)

**Keywords:** *Salvia miltiorrhiza* Bunge, seed oil, characteristics of seeds, phenolics, functional food material

## Abstract

*Salvia miltiorrhiza* seeds (SMS) are the main by-product of the production processing of *Radix Salviae Miltiorrhizae*. The main purposes of this work are to analyse the nutritional components in SMS, to explore the antioxidant activity of the chemical components in SMS and to evaluate the possibility of SMS as a raw material for functional foods. The contents of crude fibre, total protein, carbohydrates, total phenolics and flavonoids in SMS and the composition and relative content of fatty acids in SMS oil were determined. The results suggested that SMS has high contents of crude fibre (28.68 ± 4.66 g/100 g), total protein (26.65 ± 2.51 g/100 g), total phenolics (6.45 ± 0.55 mg of gallic acid equivalent/g) and total flavonoids (3.28 ± 0.34 mg of rutin equivalent/g), as well as a high level of α-linolenic acid (33.774 ± 4.68%) in their oil. Twenty-two secondary metabolites were identified in SMS residue, and nine compounds were isolated. The IC_50_ values of the total phenolic content in SMS on an ABTS radical, DPPH radical, superoxide radical and hydroxyl radical were 30.94 ± 3.68 μg/mL, 34.93 ± 4.12 μg/mL, 150.87 ± 17.64 μg/mL and 230.19 ± 24.47 μg/mL, respectively. The results indicate that SMS contain many nutrients and have high utilization value as a promising functional food.

## 1. Introduction

The genus *Salvia*, the largest member (with about 980 species) in the Lamiaceae family, is widely distributed in tropical and temperate regions of the world. Many species of this genus have important medicinal value (such as *Salvia miltiorrhiza* Bunge, *S. officinalis* L.), ornamental value (such as *S. splendens* Sellow ex Wiedneuw., *S. leucantha* CAV.) or edible value (such as *S. hispanica* L.) [1,2]. *S. miltiorrhiza* Bunge is one of the important medicinal plants of the genus *Salvia*, whose roots and rhizome are known as Danshen (*Radix Salviae Miltiorrhizae*) in the *Chinese Pharmacopoeia*. It has been used to treat cardiovascular and cerebrovascular diseases for more than 2000 years [1]. As a result of its multiple pharmacological applications, the annual consumption of *Radix Salviae Miltiorrhizae* exceeds 20 million kilograms around the world [3]. As the main production area, the planting area of *S. miltiorrhiza* in China is more than 40,000 hectares. The SMS are the main by-product of the production and processing of *Radix Salviae Miltiorrhizae*. With the increase in the planting area of *S. miltiorrhiza*, the output of SMS will continue to increase accordingly. At present, there are few research studies on the chemical constituents and activities of *Salvia miltiorrhiza* seeds (SMS). Therefore, studies on SMS are conducive to the further improvement of *S. miltiorrhiza*’s value.

The seeds of *S. hispanica* (chia) have been used as human food for 5500 years in Mexico and Guatemala [2]. The abundant existence of active polyphenols in chia seeds together with the high content of α-linolenic acid make chia seeds a superb food [4]. Residing within the same genus (*Salvia*) as chia, SMS might contain similar components as chia seeds. It is, thus, important to study the chemical composition of SMS for further development.

In this study, the physicochemical properties and chemical composition of SMS were analysed, the composition and relative contents of fatty acids (FAs) of *Salvia miltiorrhiza* seeds (SMS) oil were qualitatively measured, the secondary metabolites in SMS were investigated, the bioactive components were isolated and elucidated and the antioxidant activities were evaluated. The main purpose of the present work is to explore the nutritional composition of SMS and evaluate the feasibility of SMS as a functional food material.

## 2. Results

### 2.1. Characteristics of SMS

The value of the thousand seed weight (TSW) of SMS is 1.7402 ± 0.18 g. The total phenolic content (TPC) and the total flavonoid content (TFC) of SMS residue are 6.45 ± 0.55 mg GAE/g (mg of gallic acid equivalent) and 3.28 ± 0.34 mg RE/g (mg of rutin equivalent), respectively. The TPC and TFC in SMS are higher than that in chia seeds [5]. The value of TSW is similar to that of chia seeds [6].

The results of the proximate composition of SMS are shown in Table 1. The values of moisture and the crude ash contents of SMS are close to those of chia seeds. The crude protein content of SMS is slightly higher than that of chia seeds, while the value of the oil content is lower than that of chia seeds. There are insignificant differences in the values of carbohydrate content between SMS and chia seeds (*p* > 0.05). Nevertheless, the content of crude fibre in SMS is far lower than that in chia seeds [7].

The results above imply that the composition and content of the main nutrients in SMS are basically the same as those in chia seeds. Therefore, SMS have similar characteristics as chia seeds and thus have important development value as well. Protein, dietary fibre, carbohydrates and oil are the main nutrients in our diet. Coarse grain usually refers to the grain crops of rice, wheat, corn, soybean, barley, oats, millet, buckwheat, sorghum, broomcorn millet, quinoa, highland barley and coix seed. Brown rice is the main coarse grain that can supplement essential protein, dietary fibre, carbohydrates and other nutrient substances for the human body [8,9,10,11,12]. The contents of crude fibre, crude protein and oil in SMS are higher than those in the main coarse grains, while the carbohydrate content is lower than that in the main coarse grains (Table S2). The contents of the four main components above in SMS are relatively balanced, which indicates that SMS can also be used as a promising functional food.

The content of crude protein (26.65 ± 2.51 g/100 g) in SMS is higher than that in grape seeds and oil-tea camellia seeds but lower than that in common vegetable oil seeds, such as rapeseed, sesame, edible soybean and edible peanut [13]. The main amino acid composition of SMS protein was measured, and the results are shown in Table 2. The composition of amino acids in SMS is comprehensive and contains the essential amino acids, including lysine, tryptophan, phenylalanine, methionine, threonine, isoleucine, leucine and valine, for the human body (Table 2). Glutamic acid and arginine are the two main amino acids with the highest contents (3.14 ± 0.45 g/100 g and 2.08 ± 0.22 g/100 g, respectively) among all of the amino acids. The contents of aspartic acid, threonine, serine, glycine, tyrosine and lysine in SMS protein are higher than those of chia seeds, while the other amino acids are lower than those of chia seeds [4]. The findings above show that the content and components of the main amino acids in SMS are very similar to those of chia seeds, and SMS might be an alternative to chia seeds for good amino acid supplementation [5].

### 2.2. Characteristics of SMS Oil

The components and relative contents of the main FAs in SMS oil were determined (Figure S1), and results are presented in Table 3. The findings imply that SMS oil contains a variety of FAs with different types, and the relative contents of these FAs are very different (*p* < 0.01). Linoleic acid (LA), oleic acid (OA), α-linolenic acid (ALA), palmitic acid (PA) and stearic acid (SA) are the main constituents in all FAs. They have relative contents of 33.774 ± 4.68%, 20.139 ± 2.67%, 25.968 ± 2.96%, 8.634 ± 0.67% and 5.884 ± 0.74% of the total FAs content, respectively. The findings are consistent with the results that LA, OA, ALA, PA and SA are the main FAs in the seed oil species of Salvia [2].

ALA, LA and OA are unsaturated fatty acids (USFAs), and PA and SA are the main saturated fatty acids. The value of the relative contents of total unsaturated fatty acids (TUFAs) in SMS oil is 80.62%, which is lower than that in chia seeds (88.11%) [5]. Soybean oil, sunflower seed oil, peanut oil, corn oil, sesame oil, cottonseed oil, grape-seed oil, rapeseed oil and olive oil are the conventional edible vegetable oils in China (Table S3). The relative content of TUFAs in SMS oil is higher than those in cottonseed oil and peanut oil but lower than those in most of the conventional edible vegetable oils, such as sesame oil, soybean oil, rapeseed oil, sunflower seed oil, corn oil, grape-seed oil and olive oil (Table S3).

ALA and LA are the two most important polyunsaturated fatty acids (PUFAs) for human life and health and belong to the ω-3 and ω-6 PUFAs, respectively [13]. The content of LA in SMS oil is higher than that in soybean oil, rapeseed oil, peanut oil and olive oil and is lower than that in cottonseed oil, sunflower seed oil, corn oil, sesame oil and grape-seed oil (Table S3). The content of ALA in SMS oil is higher than that in the nine conventional edible vegetable oils in China (Table S3), which indicates that SMS oil can be used as an important ALA supplement to top up the deficiency of ALA in conventional edible oils.

The ratio of ω-3 PUFAs and ω-6 PUFAs plays an important role in human health. Studies have shown that the occurrence of many diseases is closely related to the reduction in ω-3/ω-6 PUFAs intake. The ratio of ω-3/ω-6 PUFAs in a healthy diet recommended by the World Health Organization is more than 0.2 [13]. The ratio of ω-3/ω-6 PUFAs in SMS oil (0.773) is much higher than 0.2, which indicates that the proportion of PUFAs in SMS oil is beneficial to human health. OA is a main monounsaturated fatty acid (MUFA) present in SMS oil. Compared with the nine conventional edible vegetable oils in China, the content of OA in SMS oil is relatively low; it is only higher than that in cottonseed oil and grape-seed oil and far lower than that in the other seven conventional edible vegetable oils (Table S3). The results above indicate that SMS oil has important edible value and has great potential as a new edible oil for research and development.

The higher proportion of PUFAs and the existence of abundant polyphenol active substances in chia make it a superb food [4]. The five main FAs, ALA, LA, OA, PA and SA, in chia seed oil are in the amount of 57.0%, 21.5%, 9.2%, 7.0% and 4.3%, respectively. The content of TUFAs and the ratio of ω-3/ω-6 PUFAs in chia seed oil are 88.1% and 2.7, respectively. The relative contents of PA, SA, OA and LA in SMS oil are higher than those in chia seed oil, while ALA and TUFAs contents are lower than those in chia seed oil. Therefore, the value of ω-3/ω-6 in SMS oil is far less than that in chia seed oil. Although the contents of TUFAs and the value of ω-3/ω-6 in SMS oil are lower than those in chia seed oil, the total contents of the three main USFAs (OA, LA and ALA) in SMS oil are more than 20%, which is more balanced and could meet the needs of the human body for different types of USFAs. Therefore, SMS oil has important edible value, and the seeds also have important development and utilization value as a promising functional food material.

The results of the physicochemical properties, peroxide value (POV), acid value (AV), saponification value (SV) and iodine value (IV), of SMS oil are presented in Table S4. The IV (164.58 g/100 g) of SMS oil is higher than that of the nine conventional edible vegetable oils in China (Table S4). The IV is an important index of USFAs in edible oil, and a high level of IV means a high unsaturated degree of USFAs in the oil [13]. The higher IV (164.58 g/100 g) means a higher content of USFAs in SMS oil, which is consistent with the higher content of LA (33.774%) and ALA (25.968%) found in the GC-MS determination.

The POV (3.46 meq/kg) and AV (0.35 mg/g) of SMS oil are at a low level compared with the above AV of the nine conventional edible vegetable oils in China (Table S4). The AV is an index to measure the free fatty acids content in oil and the degree of hydrolysis, and the POV is an indicator of the oxidation degree of FAs in oil. The quality and rancidity of oil can be judged by measuring the POV and AV of the oil. The lower the POV and the AV, the higher the oil quality and the lower the degree of deterioration [14]. The low values of the POV and AV imply that the quality of SMS oil is high. The SV value (202.33 mg KOH/g) of SMS oil is similar to that of most edible vegetable oils (Table S4). The data imply that the composition and content of the main USFAs in SMS oil are reasonable, and the oil has good physical and chemical properties, thus providing a new source of edible oil.

The contents of polyphenols and squalene in SMS oil are 121.24 mg/kg and 45.12 mg/kg, respectively, which is much higher than those in chia seed oil [15]. The contents of α-tocopherol, β-tocopherol, γ-tocopherol and δ-tocopherol in SMS oil are 32.45, 44.22, 576.31 and 24.66 mg/100 g, respectively. The contents of α-tocopherol and β-tocopherol in chia seed oil are higher than those in SMS oil, while the content of γ-tocopherol (652 mg/100 g) and δ-tocopherol (64.8 mg/100 g) in chia seed oil are higher than those in SMS oil [15]. The contents of campesterol, stigmasterol and sitosterol in SMS oil are 45.66 mg/100 g, 35.14 mg/100 g and 140.55 mg/100 g, respectively. The results above reveal that the fat solubility concomitant in SMS oil is at a high level, which might mean that SMS oil presents various health benefits and important nutritional value [16].

### 2.3. Identification of Secondary Metabolites in SMS

**Figure 1:**
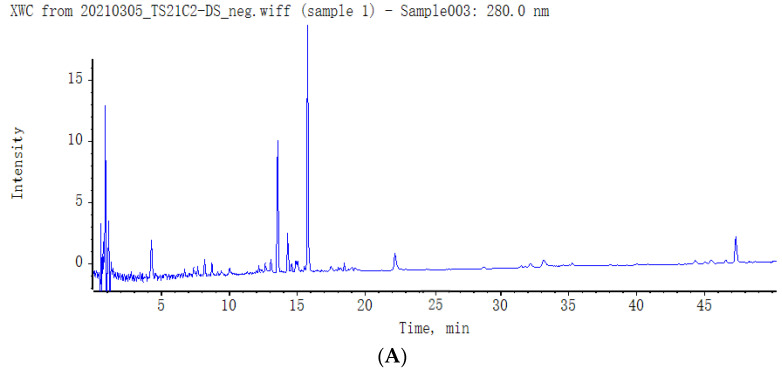

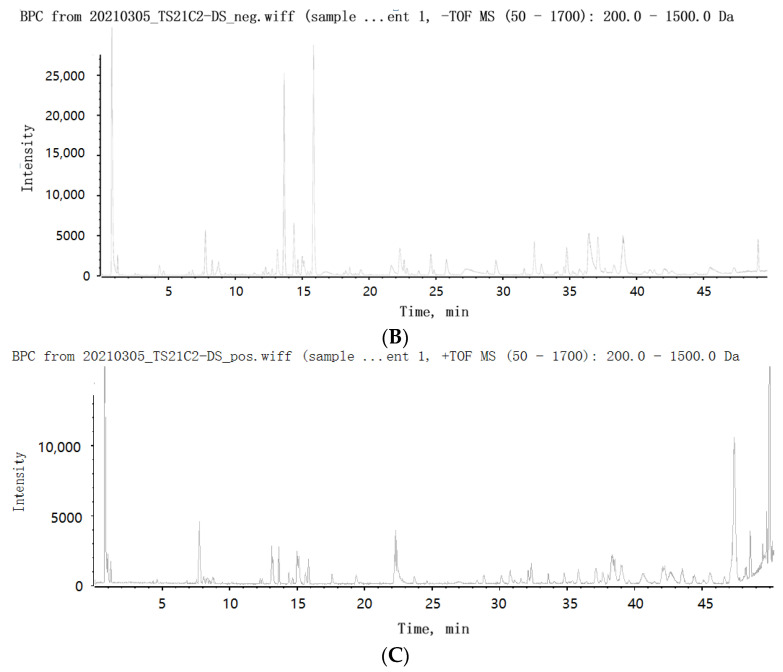
Chromatographic profile at 280 nm of the hydrolysed extract (**A**) and base peak chromatogram (BPC) of UPLC-HRMS of extract residue of *Salvia miltiorrhiza* seeds ((**B**)—negative ion mode; (**C**)—positive ion mode).

UPLC-Q-TOF-MS was employed to analyse the secondary metabolites in the extract residue of SMS. The chromatographic profile at 280 nm and the base peak chromatogram (BPC) of UPLC-HRMS of the hydrolysed extract from the extract residue of SMS are presented in Figure 1. The secondary metabolites in the extract residue of SMS were identified using the database of the GNPS platform, an in-house traditional Chinese medicine (TCM) database and the literature references. Finally, twenty-two secondary metabolites were identified (Table 4). Among them, the compounds tanshinol (**1**), albidoside (**5**), salvianolic acid E (**9**), pinoresinol glucoside (**10**), salvianolic acid B (**12**), 7‴,8‴-didehydro-salvianolic acid B (**13**), salvianolic acid L (**14**), clinopodic acid A (**15**), methyl rosmarinate (**16**) and salvianolic acid C (**17**) are phenolic acids; the compounds luteolin-7-*O*-β-d-glucoside (**7**), luteolin (**18**) and apigenin (**19**) are flavonoids; the compounds asiatic acid (**20**), dihydrotanshinone I (**21**) and cryptotanshinone (**22**) are terpenoids; the compound pinoresinol glucoside (**10**) is a lignin; the compound albidoside (**5**) is an iridoid glycoside; and the compound tuberonic acid glucoside (**3**) is a tuberonic acid glucoside. The detailed identification process of compounds is shown in the Supporting Information (Text S1).

### 2.4. Structure Identification of the Isolated Compounds

Nine compounds were isolated via total phenolic extraction (TPE) and were elucidated as salviaflaside (**1**) [22], rosmarinic acid (**2**) [25], cryptotanshinone (**3**) [29], dihydrotanshinone I (**4**) [29], apigenin (**5**) [31], luteolin (**6**) [31], luteolin-7-*O*-β-d-glucoside (**7**) [32], tanshinol (**8**) [17] and salvianolic acid B (**9**) [25]. All of the compounds were first isolated from SMS. The spectral data of the isolated compounds are shown in the Supporting Information (Text S2).

### 2.5. Antioxidant Activity of TPE

The inhibitory percentages of TPE on an ABTS radical, DPPH radical, superoxide radical and hydroxyl radical were assessed by colorimetric method, and vitamin C (Vc) was used as the positive control. It can be seen from Figure S2 that with the increase in TPE concentration, the inhibition effect on the four free radicals showed good concentration dependence. With concentrations ranging from 5.0 μg/mL to 60.0 μg/mL, the scavenging abilities of TPE on the ABTS radical and DPPH radical ranged between 8.35 and 75.8% and 8.3 and 69.8%, respectively. The scavenging abilities of TPE on the superoxide radical and hydroxyl radical ranged between 26.8 and 84.6% and 14.6 and 75.8% with the TPE concentration increased from 50 μg/mL to 400 μg/mL. The IC_50_ values of TPE on the ABTS radical, DPPH radical, superoxide radical and hydroxyl radical were 30.94 ± 3.68 μg/mL, 34.93 ± 4.12 μg/mL, 150.87 ± 17.64 μg/mL and 230.19 ± 24.47 μg/mL, respectively, while the IC_50_ values of Vc on the four radicals were 23.31 ± 2.65 μg/mL, 28.03 ± 3.74 μg/mL, 100.95 ± 9.56 μg/mL and 139.48 ± 12.97 μg/mL, respectively. The IC_50_ values of TPE on the radicals were more than that of Vc, indicating that the antioxidant activity of TPE was less than that of Vc. The results reveal that the antioxidant activity of SMS extract is lower than that of chia seed extract [7,33].

## 3. Discussion

The results suggest that the contents of crude fibre, crude protein and oil in SMS are higher than those in the main coarse grains, while the carbohydrate content is lower than that in the main coarse grains, which indicates that SMS could also be used as a promising functional food. The composition of amino acids in SMS is comprehensive; the contents of aspartic acid, threonine, serine, glycine, tyrosine and lysine of SMS protein are higher than those in chia seeds, while the other amino acids are lower than those in chia seeds [4], and the essential amino acids are higher than chia seeds from Kenya [34]. The composition and content of fatty acids in SMS oil are reasonable. The value of the relative contents of TUFAs in SMS oil is lower than that in chia seeds [5]. The ratio of ω-3/ω-6 PUFAs in SMS oil (0.773) is much higher than 0.2, which indicates that the proportion of PUFAs in SMS oil is beneficial to human health, and the oil has good physical and chemical properties, which indicates that SMS oil has important edible value. The TPE from SMS residue had good antioxidant activity in vitro. The chemical constituent study of the TPE from SMS residue led to the isolation and identification of nine phenolics, including salviaflaside (**1**), rosmarinic acid (**2**), cryptotanshinone (**3**), dihydrotanshinone I (**4**), apigenin (**5**), luteolin (**6**), luteolin-7-*O*-β-d-glucoside (**7**), tanshinol (**8**) and salvianolic acid B (**9**). As dietary bioactive substances, phenolics, including simple phenols and their derivatives, and flavonoids and their derivatives show a variety of functions and biological activities [33]. Polyphenols are considered to be powerful antioxidants, which have functions including inhibiting the oxidative deterioration of food, protecting the human body from oxidative stress diseases, and so on [33]. Salviaflaside (**1**), rosmarinic acid (**2**), cryptotanshinone (**3**), dihydrotanshinone I (**4**), tanshinol (**8**) and salvianolic acid B (**9**) are common compounds in *Radix Salviae Miltiorrhizae*, and they are also the active components isolated firstly from SMS [35]. *Radix Salviae Miltiorrhizae* is not only a traditional Chinese medicine but also a food that has good edible value. Similar to the secondary metabolites of *Radix Salviae Miltiorrhizae*, SMS also have certain edible value.

The IC_50_ values of the TPE in SMS on an ABTS radical, DPPH radical, superoxide radical and hydroxyl radical were 30.94 ± 3.68 μg/mL, 34.93 ± 4.12 μg/mL, 150.87 ± 17.64 μg/mL and 230.19 ± 24.47 μg/mL, indicating that SMS have antioxidant activity.

Studies revealed that rosmarinic acid (**2**), apigenin (**5**), luteolin (**6**) and tanshinol (**8**) were also identified in chia seeds [5,33], which implies that caffeoyl derivatives, flavonoids and organic acids are essential components in SMS as they are in chia seeds. Salviaflaside (**1**) and rosmarinic acid (**2**) were proven to have good ABTS radical and DPPH radical scavenging activities [36]; apigenin (**5**) [37] and luteolin (**6**) [28] proved to have good DPPH radical scavenging activity, and tanshinol (**8**) was found to have a better protective effect on the injury of rat cardiac mitochondria caused by hydroxyl free radicals [38]. Results revealed that salvianolic acid B (**9**) presented a strong scavenging capacity against DPPH, superoxide and hydroxyl radicals in a concentration-dependent manner [39]. The studies above imply that the isolated compounds are the main bioactive components in SMS residue and that SMS have good edible value.

## 4. Materials and Methods

### 4.1. Plant Materials

The SMS were collected from Luoning County, Luoyang, Henan Province, China, in October 2018. The sample was authenticated by Xiao-gai Hou, a botany professor in agriculture. The voucher specimens (2023018) were deposited in the Specimens Hall of Natural Resources of the Funiu Mountains at the Henan University of Science and Technology.

### 4.2. Chemicals

The analytical or HPLC grade chemicals, such as methanol, chloroform, hexane, petroleum ether, ethyl acetate, and so on, were obtained from Luoyang Weiyue Chemical Glass Co., Ltd. (Luoyang, China). Acetonitrile, methanol and formic acid, LC-MS grade, were purchased from Merck (Shanghai, China).

### 4.3. Characterization of SMS

The SMS were dried to a constant weight, and the TSW (g) was determined by the national standard method of the People’s Republic of China [40] (GB/T 5519-88). The SMS were crushed, and then the oil was extracted with a Soxhlet apparatus. The method described in the literature was used to calculate the oil content [41].

The residue of the SMS was collected and dried at 110 °C for 2 h. The International Organization for Standardization (ISO) methods were used to determine the proximate composition of the SMS: moisture and volatile matter [42] (ISO 771: 1977), crude fibre [43] (ISO 7716865: 2000), total protein [44] (ISO 20483: 2006) and total ash [45] (ISO 749: 1977). The contents of carbohydrates were determined as the difference, using the following equation:

The content of carbohydrates = [100 − (% moisture + % ash + % proteins + % lipids)]

The contents of total phenolics and total flavonoids in the SMS were determined by colorimetric methods. The TPC was evaluated by the method presented in the literature [7], and the TFC was measured using the detailed information presented in the literature [46]. The TPC and TFC were recorded as mg of gallic acid equivalent (mg GAE·g^−1^) and rutin equivalent (mg RE·g^−1^) of SMS, respectively.

### 4.4. Characterization of SMS Oil

The profile of FAs in SMS oil were analysed by GB/T methods [47,48] (GB/T 17376-2008 and 17377-2008). The components were analysed with a GC-MS instrument (Agilent 7890A-5975, Agilent Technology Co., Ltd., Beijing, China) equipped with Agilent Chem Station E2.00 software, and using the methods and chromatographic conditions presented by Liu et al. [14]. The composition of the main FAs was determined based on the corresponding reference substance, and the content of each component was calculated from the chromatographic peak area by a computerized integrator.

The ISO methods were employed to determine the physicochemical properties of SMS oil: AV [49] (ISO 660, 1996), POV [50] (ISO 3960, 2001), SV [51] (ISO 3657, 2002) and IV [52] (ISO 3961, 1996).

The contents of micronutrients in SMS oil were also determined: polyphenols [53] (LS/T 6119-2017), squalene [54] (LS/T 6120-2017), vitamin E [55] (GB 5009.82-2016) and phytosterols [56] (GB/T 25223-2010).

### 4.5. The Investigation of the Secondary Metabolites in SMS

#### 4.5.1. The Sample Preparation

The residue of SMS (1 g) was extracted with 25 mL of 70% ethanol for 30 min by an ultrasonic device (Ymnl-2008DE, Nanjing Immanuel Equipment Co., Ltd., Nanjing, China) at 300 W, 40 KHz. The extraction was cooled to room temperature and centrifuge equipment (LD5-2B, Beijing Jingli Centrifuge Co., Ltd., Beijing, China) was used at 12,000 rpm for 5 min, and the supernatant was obtained for UPLC-Q-TOF/MS analysis.

#### 4.5.2. UPLC-Q-TOF/MS Analysis

Agilent 1290 UPLC (Agilent Technology Co., Ltd., Beijing China) was used for analysis. Chromatographic column: an Agilent ZORBAX RRHD Eclipse XDB-C18 (2.1 × 100 mm, 1.8 µm); column temperature: 30 °C; injection volume: 1 μL; detection wavelength: 190–400 nm; flow rate: 0.3 mL/min; mobile phase ratio: 0.1% formic acid (A)-acetonitrile (B); elution gradient: 0 min (B, 5%) → 2 min (B, 5%) → 30 min (B, 40%) → 35 min (B, 50%) → 45 min (B, 55%) → 50 min (B, 95%) → 52 min (B, 95%) → 54 min (B, 5%).

Detection mode of UPLC-Q-TOF/MS (SYNAPT G2-Si HDMS, Waters Technology Co., Ltd., Shanghai, China): ESI ion source negative/positive ion mode. The mass spectrum parameters are shown in Table S1.

### 4.6. The Extraction and Isolation of Phenolic Compounds from TPE

The total phenolic extraction (TPE) in SMS was performed in this study. The SMS residue (5 kg) was extracted under reflux with 90% ethanol as solvent for 5 h. The solvent in the extract was recovered by vacuum distillation, and the crude extract (260 g) was then obtained. The 200 g crude extract was dispersed in distilled water (8000 mL), mixed thoroughly and subsequently filtered by filter papers, and then 10 L polyamide resin column (D-101) was added into the filtrate for adsorption. After adsorption equilibrium was reached, the resin was loaded onto a glass column and was washed by distilled water and then desorbed with 10%, 30%, 50%, 70% and 90% ethanol (*v*/*v*) at a flow rate of 2.0 BV/h, respectively. The desorbed fraction was concentrated under reduced pressure. The dried extraction of each fraction was collected, and the contents of the total phenolics were calculated. As the result of the highest content of total phenolic compound contained (TPE), the 50% fraction (22 g) was collected for further antioxidant activity testing and chemical component separation and identification.

The TPE (20 g) of the SMS residue was dissolved in methanol and filtered with filter papers, and the filtrate was mixed with 30 g silica gel. The mixture was dried and then chromatographed over silica gel (1000 g) column chromatography (CC) using a gradient elution with a CHCl_3_-CH_3_OH solvent system (1:0, 15:1, 13:1, 9:1, 7:1, 5:1, 3:1 and 1:1), and fifteen fractions (fraction 1–fraction 15) were obtained.

**Figure 2:**
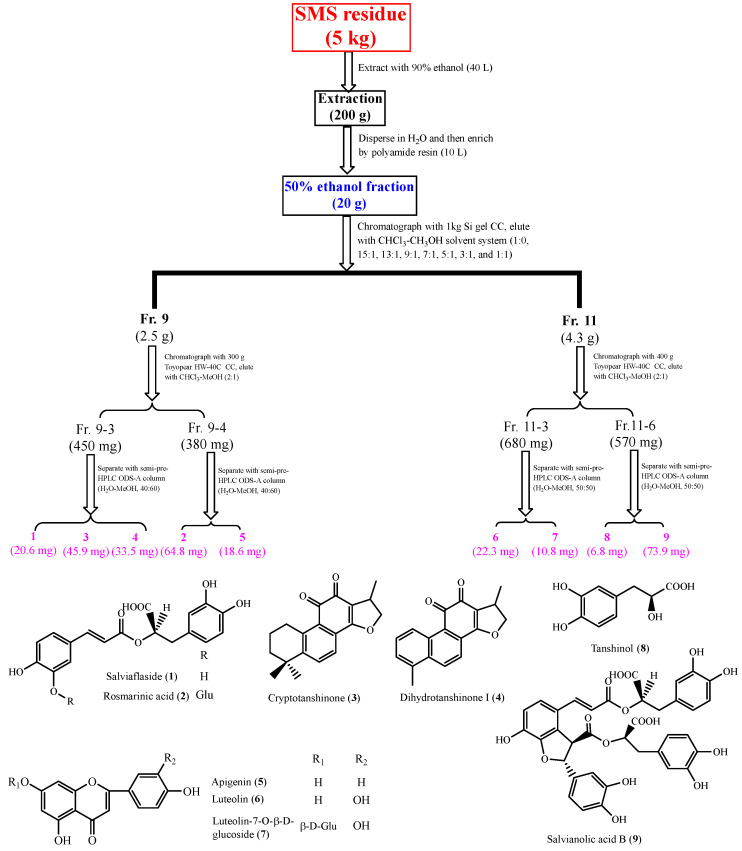
The separation process and chemical structures of compounds **1**–**9**.

Fraction 9 (2.5 g) and fraction 11 (4.3 g) were chromatographed on a Toyopearl HW-40C CC and then were purified by a semi-pre-HPLC ODS-A column [H_2_O-MeOH], respectively, and compounds **1**–**9** were obtained. The specific separation process of the compounds is shown in Figure 2.

### 4.7. Antioxidant Capacity

The free radical scavenging activities of TPE from the SMS residue were evaluated by inhibiting an ABTS radical, DPPH radical, superoxide radical and hydroxyl radical using colorimetric methods described in the literature [46].

### 4.8. Data Analysis

All experiments were carried out in triplicate (*n* = 3). The results are expressed as the mean ± standard deviation (SD). Very significant (*p* < 0.01) and significant (0.01 < *p* < 0.05) are used in this paper. Statistical analysis was performed with one-way ANOVA.

## 5. Conclusions

In this study, the proximate composition, phytochemical components and antioxidant activity of SMS were evaluated. The results showed that the oil yield, composition and relative contents of the main FAs in SMS oil were basically the same as those of chia seed. The relatively high content of the TUFAs, along with the high (ω-3)/(ω-6) value implies that SMS oil might become a healthy edible oil. The high content of crude fibre, along with the high plant protein content and the comprehensive composition of the amino acids in SMS, revealed that SMS might be an alternative to chia seeds for good amino acid supplementation. Twenty-two secondary metabolites were identified from the SMS residue, including thirteen phenolic acids, three flavonoids, two diterpenoids, one triterpenoid, one lignin, one iridoid glycoside and one other type of compound. Nine compounds, including terpenoids, flavonoids and plant polyphenols, were obtained from the TPE of the SMS residue, and all of the compounds were isolated firstly from SMS. Rich structural features of the isolated compounds and the antioxidant activity of the TPE on radicals also imply that SMS have good edible and health value. The multiple pharmacological effects of *Radix Salviae Miltiorrhizae* led to more than 40,000 hectares of the planting area of *S. miltiorrhiza* in China. The SMS are the main by-product of the production process of *Radix Salviae Miltiorrhizae*. With the increase in planting area of *S. miltiorrhiza*, the output of seeds will continue to increase. Therefore, SMS will become a promising functional food material.

## Figures and Tables

**Table 1:** The main constituents in *Salvia miltiorrhiza* seeds (g/100 g) (*n* = 3, x ± s).

Seeds	*S. miltiorrhiza*
Crude protein	26.65 ± 2.51
Crude fibre	28.68 ± 4.66
Oil content	28.45 ± 3.44
Moisture	4.26 ± 0.65
Crude ash	3.69 ± 0.37
Carbohydrate	34.62 ± 3.64

**Table 2:** Amino acid profile of *Salvia miltiorrhiza* seeds (g/100 g) (*n* = 3, x ± s).

Amino Acid	*S. miltiorrhiza*	Amino Acid	*S. miltiorrhiza*
Aspartic acid	1.72 ± 0.15	Isoleucine	0.74 ± 0.08
Threonine	0.81 ± 0.10	Leucine	1.24 ± 0.15
Serine	1.14 ± 0.11	Tyrosine	0.97 ± 0.12
Glutamic acid	3.14 ± 0.45	Phenylalanine	0.89 ± 0.12
Glycine	1.05 ± 0.13	Lysine	1.05 ± 0.08
Alanine	0.87 ± 0.08	Histidine	0.46 ± 0.04
Cysteine	0.35 ± 0.04	Arginine	2.08 ± 0.22
Valine	0.93 ± 0.09	Proline	0.68 ± 0.05
Methionine	0.52 ± 0.05	Tryptophan	0.46 ± 0.03

**Table 3:** Fatty acid profile of *Salvia miltiorrhiza* seeds (*n* = 3, x ± s).

Fatty Acid Composition	Content (%)	Fatty Acid Composition	Content (%)
Dodecanoic acid (C12:0)	0.01 ± 0.01	Linoleic acid (C18:2) (ω-6)	33.774 ± 4.68
Myristic acid (C14:0)	0.113 ± 0.01	Linolenic acid (C18:3) (ω-3)	25.968 ± 2.96
Pentadecanoic acid (C15:0)	0.037 ± 0.01	18-Methylnonadecanoic acid (C19:0)	0.478 ± 0.01
Cis-5-dodecenoic acid (C12:1)	0.012 ± 0.01	Cis-13-Eicosenoic acid (C20:1)	0.511 ± 0.11
Palmitic acid (C16:0)	8.634 ± 0.67	9,11,13,15-Octadecatetraenoic acid (C18:4) (ω-3)	0.013 ± 0.01
E-9-Hexadecenoic acid (C16:1)	0.148 ± 0.06	ω-3 fatty acid	26.098 ± 2.97
Heptadecanoic acid (C17:0)	0.145 ± 0.08	ω-6 fatty acid	33.774 ± 4.68
Cis-10-Heptadecenoic acid (C17:1)	0.064 ± 0.01	(ω-3)/(ω-6)	0.773
16-methyl-Heptadecanoic acid (C18:0)	0.094 ± 0.03	Total fatty acid	95.879
Stearic acid (C18:0)	5.884 ± 0.86	TUFAs ^a^	80.62
Oleic acid (C18:1)	20.139 ± 2.67		

^a^ TUFAs: the total unsaturated fatty acids.

**Table 4:** Characterization of compounds by UPLC-Q-TOF/MS from the seed cake of *Salvia miltiorrhiza*.

No.	RT (min)	Compound	Adduct Ions	Class	Found *m/z*	Expected *m/z*	Error (ppm)	Formula	Major Fragments	References
1	2.86	Tanshinol	[M − H]^−^	Phenolic acids	197.0454	197.0455	−0.7	C_9_H_10_O_5_	179.0350, 162.8392, 135.0472, 123.0465, 72.9933	[17]
2	8.27	p-Hydroxycinnamic acid sophoroside	[M − H]^−^	Phenolic acids	487.1458	487.1457	0.2	C_21_H_28_O_13_	487.1515, 265.0692, 205.0507, 163.0405, 145.0300	GNPS; TCM
3	8.74	Tuberonic acid glucoside	[M − H]^−^	Other	387.1659	387.1661	−0.4	C_18_H_28_O_9_	387.1625, 207.0936, 163.1129	[18]
4	11.42	Salvianolic acid H	[M − H]^−^	Phenolic acids	537.1047	537.1039	1.6	C_27_H_22_O_12_	339.0464, 295.0487, 267.0722, 229.0140	GNPS
5	12.27	Albidoside	[M − H]^−^	Iridoid glycosides	521.1681	521.1665	−0.5	C_25_H_30_O_12_	521.1694, 503.1556, 325.0907, 265.0707, 205.0491	[19]
6	12.75	Salvinoside	[M − H]^−^	Phenolic acids	879.1983	879.1989	−0.7	C_42_H_40_O_21_	879.1907, 717.1536, 699.1454, 519.0918, 475.0770, 399.0467	[20]
7	13.15	Luteolin 7-*O*-β-d-glucoside	[M − H]^−^	Flavonoids	447.0933	447.0933	−0.4	C_21_H_20_O_11_	447.0932, 285.0379	[21]
8	13.65	Salviaflaside	[M − H]^−^	Phenolic acids	521.1300	521.1301	−0.1	C_24_H_26_O_13_	521.1264, 359.0740, 323.0750, 161.0238, 135.0439	[22]
9	14.39	Salvianolic acid E	[M − H]^−^	Phenolic acids	717.1467	717.1461	0.8	C_36_H_30_O_16_	717.1377, 519.0900, 475.0994, 339.0475, 321.0375, 243.0286, 197.0465, 109.0299	[23]
10	14.68	Pinoresinol glucoside	[M − H]^−^	Lignin	535.1821	535.1815	0.6	C_26_H_32_O_12_	535.1818, 373.1288, 355.1191, 295.1067, 179.0550	[24]
11	15.85	Rosmarinic acid	[M − H]^−^	Phenolic acids	359.0773	359.0772	0.2	C_18_H_16_O_8_	359.0742, 197.0441, 179.0343, 161.0238, 135.0446, 123.0442, 72.9927	[25]
12	16.60	Salvianolic acid B	[M − H]^−^	Phenolic acids	717.1469	717.1461	1.1	C_36_H_30_O_16_	717.1342, 519.1010, 475.0997, 321.0416	[23]
13	17.59	7‴,8‴-Didehydro-salvianolic acid B	[M − H]^−^	Phenolic acids	715.1301	715.1305	−0.5	C_36_H_28_O_16_	715.1297	GNPS; TCM
14	17.68	Salvianolic acid L	[M − H]^−^	Phenolic acids	717.1470	717.1461	1.2	C_36_H_30_O_16_	519.1012, 321.0432	[23]
15	18.26	Clinopodic acid A	[M − H]^−^	Phenolic acids	343.0826	343.0823	0.8	C_18_H16O7	191.0428, 181.0517, 161.0238, 135.0452, 119.0515	[26]
16	18.86	Methyl rosmarinate	[M − H]^−^	Phenolic acids	373.0928	373.0929	−0.2	C19H_18_O_8_	197.0423, 179.0397, 175.0398, 160.0170, 135.0448, 123.0456	[27]
17	18.99	Salvianolic acid C	[M − H]^−^	Phenolic acids	491.0991	491.0989	1.1	C_26_H_20_O_10_	491.1027, 311.0567, 267.0635, 135.0465	[23]
18	19.38	Luteolin	[M − H]^−^	Flavonoids	285.0399	285.0405	−0.6	C_15_H_10_O_6_	285.0382, 175.0397, 149.0249, 133.0297	[21]
19	22.31	Apigenin	[M − H]^−^	Flavonoids	269.0454	269.0455	−0.5	C_15_H_10_O_5_	269.0425, 225.0518, 183.0548, 161.0242, 149.0240, 117.0348	[21]
20	34.08	Asiatic acid	[M − H]^−^	Triterpenoids	487.3424	487.3429	−1	C_30_H_48_O_5_	487.3437, 469.3293	[28]
21	35.54	Dihydrotanshinone I	[M + H]^+^	Diterpenoids	279.1014	279.1016	−0.6	C_18_H_14_O_3_	279.0966, 203.0817, 189.0682, 149.0217, 121.0254	[29]
22	40.72	Cryptotanshinone	[M + H]^+^	Diterpenoids	297.1486	297.1485	0.3	C_19_H_20_O_3_	297.1516, 255.0994, 236.0855	[30]

RT, retention time.

## Data Availability

Data are contained within the article and Supplementary Materials.

## Supplementary Materials

The following supporting information can be downloaded at: https://www.mdpi.com/article/10.3390/molecules29071483/s1, Figure S1: Fatty acid profile in seeds oil of S. miltiorrhiza; Figure S2: Effects of TPE on the radical scavenging rates; Figure S3: Possible mass fragmentation pathways of tanshinol, salvinoside, salvianolic acid B and salvianolic acid E; Figure S4: Possible mass fragmentation pathways of rosmarinic acid, salviafliside, methyl rosmarinate, salvianolic acid C and salvianolic acid H; Figure S5: Possible mass fragmentation pathways of p-hydroxycinnamic acid sophoroside and clinopodic acid A; Figure S6: Possible mass fragmentation pathways of apigenin, luteolin and luteolin-7-O-β-d-glucoside; Figure S7: Possible mass fragmentation pathways of asiatic acid, dihydrotanshinone I and cryptotanshinone; Figure S8: Possible mass fragmentation pathways of tuberonic acid glucoside, albidoside and pinoresinol glucoside; Table S1: Mass parameters of sciex triple TOF; Table S2: The main constituents in coarse grains; Table S3: Common fatty acid composition of some conventional edible vegetable oils; Table S4: Physicochemical properties of SMS oil and some vegetable oils; Text S1: Identification of secondary metabolites in SMS; Text S2: The spectral data of the isolated compounds. References [57,58,59,60,61,62,63,64,65,66,67,68,69,70] are cited in the Supplementary Materials.

## Abbreviations

The following abbreviations are used in this manuscript:

SMS	*Salvia miltiorrhiza* seeds
FA	fatty acid
TSW	thousand seed weight
TPC	total phenolic content
TFC	total flavonoid content
GAE	gallic acid equivalent
RE	rutin equivalent
LA	linoleic acid
OA	oleic acid
ALA	α-linolenic acid
PA	palmitic acid
SA	stearic acid
USFA	unsaturated fatty acid
TUFA	total unsaturated fatty acid
PUFA	polyunsaturated fatty acid
MUFA	monounsaturated fatty acid
TPE	total phenolic extraction
POV	peroxide value
AV	acid value
SV	saponification value
IV	iodine value
