# Supplementary material for: An Evaluation of the Edible Value of Salvia miltiorrhiza Seeds: Proximate Composition, Phytochemical Components and Antioxidant Activity
Source: Molecules. 2024 Mar 27;29(7):1483. doi: 10.3390/molecules29071483 (PMC11012964; doi:10.3390/molecules29071483)
Supplement: Supplementary file 1 [file molecules-29-01483-s001.zip › molecules-2905197-supplementary.pdf]

Supporting information

An Evaluation of the Edible Value of *Salvia miltiorrhiza* Seeds: Proximate Composition, Phytochemical Components and Antioxidant Activity

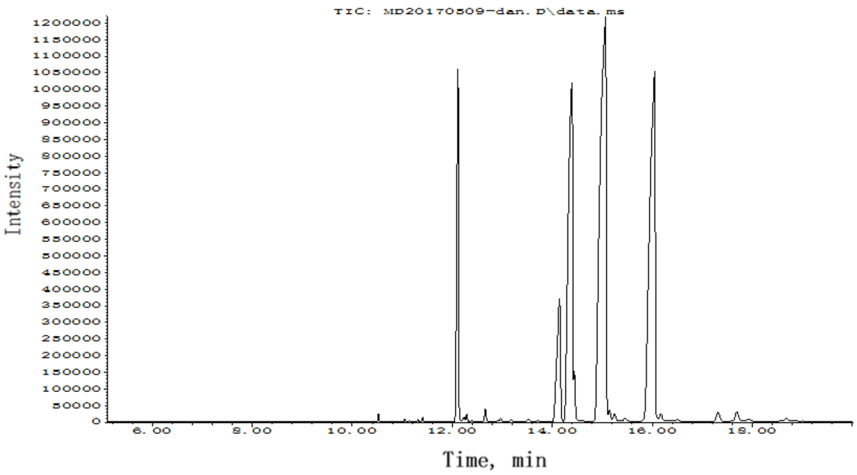

Fig. S1. Fatty acid profile in seeds oil of *S. miltiorrhiza*.

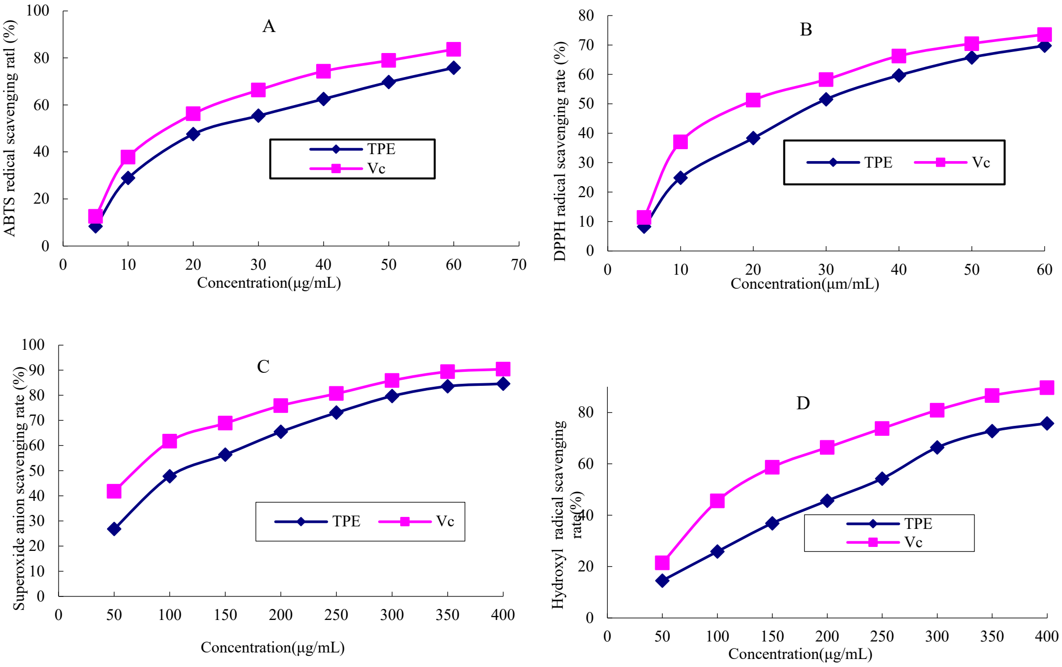

Fig. S2 Effects of TPE on the radical scavenging rates

Table S1 Mass parameters of sciex triple TOF

MS parameter	Values	MS/MS parameter	Values
TOF mass range	50-1700	MS/MS mass range	50-1250
Ion Source Gas 1 (psi)	50	Declustering Potential (V)	100
Ion Source Gas 2 (psi)	50	Collision Energy (eV)	±40
Curtain Gas (psi)	35	Collision Energy Spread (eV)	20
Ion Spray Voltage Floating (V)	-4500/5000	Ion Release Delay (ms)	30
Ion Source Temperature (°C)	500	Ion Release Width (ms)	15
Declustering Potential (V)	100		
Collision Energy (eV)	10		

Table S2 The main constituents in coarse grains.

Coarse grain	Crude protein	Crude fibre	Carbohydrate	Crude Oil
Barley	10.5	9.9	66.0	2.2
Oats	16.9	10.6	66.2	6.9
Millet	9.0	1.6	73.5	3.1
Buckwheat	9.3	6.5	66.5	2.3
Sorghum	10.4	4.3	70.4	4.3
Broomcorn millet	14.7	4.0	70.0	3.6
Quinoa	13.1	5.9	68.9	5.8
Highland barley	8.1	3.1	75.0	1.5
Coix seed	12.8	2.0	71.1	3.3
Brown rice	8.2	1.5	81.6	2.1

Values in **Table S2** were taken from literatures (Wang et al., 2016; Huang et al., 2016; Zhang et al., 2020; Hou & Shen, 2020; Chen & Liao, 2020; Liu et al., 2020b; Tian et al., 2019; Li et al., 2019).

Table S3 Common fatty acid composition of some conventional edible vegetable oils

Oils	PA ($C_{16:0}$)	SA ($C_{18:0}$)	OA ($C_{18:1}$)	LA ($C_{18:2}$)	ALA ($C_{18:3}$)	TCMUFA	(ω-3)/(ω-6)
Olive oil	13.51	4.46	72.71	6.07	0.72	81.33	0.12
Cottonseed oil	26.55	2.42	10.12	60.12	0.11	70.32	0.0018
Soybean oil	12.45	4.91	26.38	17.91	6.95	81.17	0.22
Rapeseed oil	4.82	2.10	48.68	17.92	8.67	91.60	0.48
Sunflower seed oil	7.97	4.87	22.54	62.18	0.21	85.36	0.0032
Peanut oil	12.60	5.14	42.24	31.37	0.11	74.90	0.0035
Corn oil	13.76	2.21	29.67	51.70	1.02	83.02	0.020
Sesame oil	9.50	5.13	40.22	45.11	0.58	85.60	0.013
Grape Seed Oils	7.28	3.26	8.06	80.84	0.28	90.21	0.0035

[a]- Values were taken from literatures (Liu et al., 2020; Taghvaei et al., 2014; Hui et al., 2006; Zhang et al., 2015; Gao et al., 2009; Yu et al., 2019; Gan et al., 2018; Zhang et al., 2019).

Table S4 Physicochemical properties of SMS oil and some vegetable oils

Oils	IV[a]	AV	SV	POV
S. miltiorrhiza seeds oil	164.58	0.35	202.33	3.46
Olive oil [b]	87.13	3.14	188.79	6.4
Cottonseed oil	103-115	<1.0	191-199	5.0
Soybean oil	130.7	3.3	192.3	5.2
Rapeseed oil	134.3	0.78	201.7	3.48
Sunflower seed oil	89.0	1.5	192.0	<6.0
Peanut oil	108.0	0.25	183.2	2.85
Corn oil	103-135	4.0	187-195	<6
Sesame oil	104-120	1.18	186-195	1.86
Grape Seed Oils	130.04	5.06	193.6	6.3

[a] - IV-iodine value, AV-acid value, SV-saponification value and POV-peroxide value.

[b] -Values were taken from literatures (Zhang et al., 2015; Gao et al., 2009; Yu et al., 2019; Gan et al., 2018; Zhang et al., 2019).

Text S1. **Identification of secondary metabolites in SMS**

1. Phenolic acids

A total of 13 polyphenolic acids were identified from SMS meal, including compounds **1**, **3**, **5**, **7**, **9-10** and **12-18**. Phenolic acids are one of the characteristic components, and widely found in Radix *Salviae Miltiorrhizae* (Shan et al., 2021). From the research results, the spectral characteristics of polyphenols in SMS meal are consistent with those reported in Radix *Salviae Miltiorrhizae*.

In the negative ion mode, compound **1** presented precursor ion at m/z 197.0454 $[M-H]^-$, and the corresponding molecular formula was $C_9H_9O_5$. The fragment ions m/z 179.0350, m/z 135.0472 and m/z 123.0465 were also obtained. It implied that compound **1** was easy to lose neutral molecules CO, CO_2, H_2O and CH_2O_2 under the action of ionization, thus there was carboxyl group in the structure of compound **1** (Yang et al., 2019). The molecular weight and fragment m/z values, combined with the literature results revealed that compound **1** was tanshinol (Mei et al., 2018). The possible cracking pathway of compound **1** was shown in **Figure S3**.

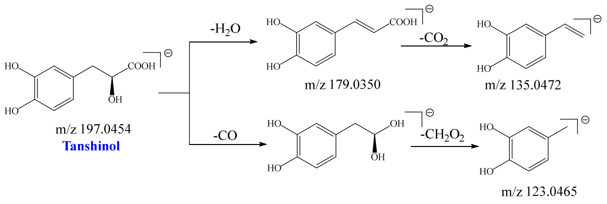

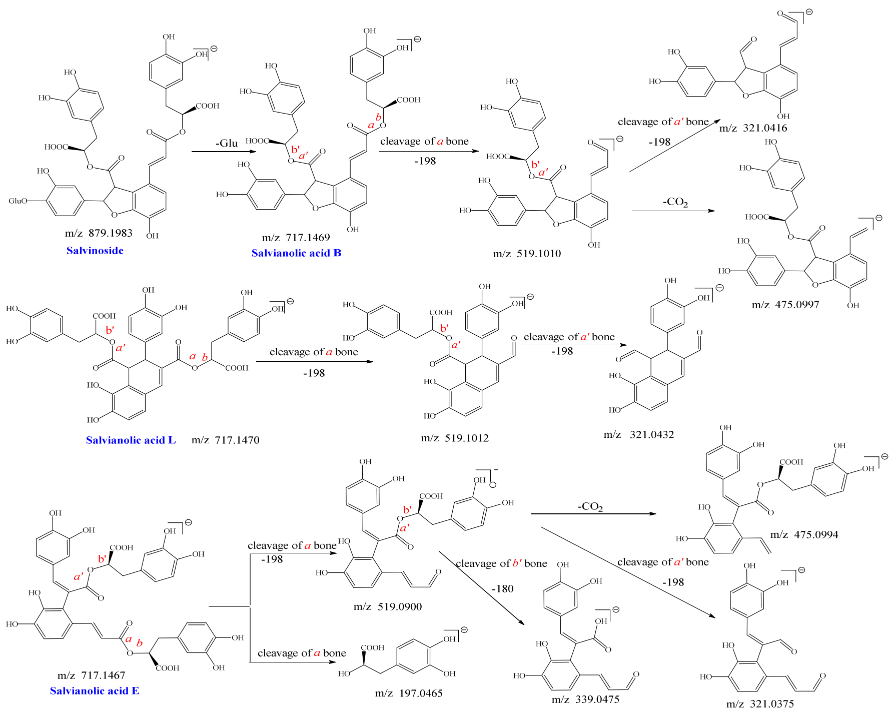

Fig. S3 Possible mass fragmentation pathways of tanshinol, salvinoside, salvianolic acid B and salvianolic acid E

In ESI negative ion mode, the quasi-molecular ion peaks m/z of compound **9**, **12** and **14** were all 717.1470 $[M-H]^-$, and the corresponding molecular formula was $C_{36}H_{30}O_{16}$. Moreover, it could be seen from the MS fragments that these three compounds had the same fragment ions m/z 519.1012 and m/z 321.0432, indicating that the second-order mass spectrum cracking laws of compounds **9**, **12** and **14** were similar, and these three compounds might be isomers. Combined with the structural characteristics of polyphenols in Radix *Salviae Miltiorrhizae*, it could be seen that fragment ions m/z 519.1012 $[M-H-198]^-$ and m/z 321.0432$[M-H-198-198]^-$ were obtained after the loss of two tanshinol unit (-198), indicating that these compounds had two tanshinol structural fragments, which was consistent with the neutral loss of phenolic compounds, and the characteristic fragments conformed to the structural

characteristics of polymeric polyphenols of Radix *Salviae Miltiorrhizae* (Liu et al., 2007). The mass spectrum characteristics of compound **9** were consistent with those of salvianolic acid L reported in literature (Liu et al., 2007), thus the compound was determined as salvianolic acid L. In addition to the fragment ions above, compound **14** also had fragment ions such as m/z 197.0465, m/z 339.0475 and m/z 475.0994, which were tanshinol structural fragment, and the remaining fragment ions after losing tanshinol fragments in different ways, respectively. The mass spectrum information, combined with data reported in the literature (Liu et al., 2007), identified compound **14** as salvianolic acid E. The fragment ions, combined with the structural characteristics and search results revealed that the compound **12** was salvianolic acid B (Liu et al., 2007). Compound **6** and compound **12** produced the same ion m/z 717.1470, it could thus be concluded that they might be the same type of components. The analysis of fragment ions in ESI/MS^n mass spectrometry of compound **6** implied that the precursor ion m/z 879.1983 and fragment ion m/z 717.1536 were parent-child relationship, and the fragment ion (m/z 717.1536) was obtained as the loss of a neutral fragment (dehydrated hexosyl) with a mass of 162 by the precursor ion (m/z 879.1983), indicating that compound **6** had a fragment of glucose. It could be seen that compound **6** was produced by the cleavage of glycosidic bond of compound **12**. According to relevant reference (Deng et al., 2008), compound **6** was salvinoside.

The quasi-molecular ion peak of compound **4** in negative ion mode was 537.1047 $[M-H]^-$, and the fragment ions were m/z 339.0464, 295.0487, 267.0722 and 229.0140. Among them, fragment ion m/z 339.0464 was the residual ion $[M-198-H]^-$,

and ions m/z 295.0487 and m/z 267.0722 were fragments of fragment ion (m/z 339.0464) after losing neutral molecules CO_2 and CO, which conformed to the structural characteristics of polymeric polyphenols in phenolic acids (Liu et al., 2007). Combined with GNPs platform, compound **4** was identified as salvianolic acid H, and the mass spectrum cleavage pathway was shown in **Figure S3**.

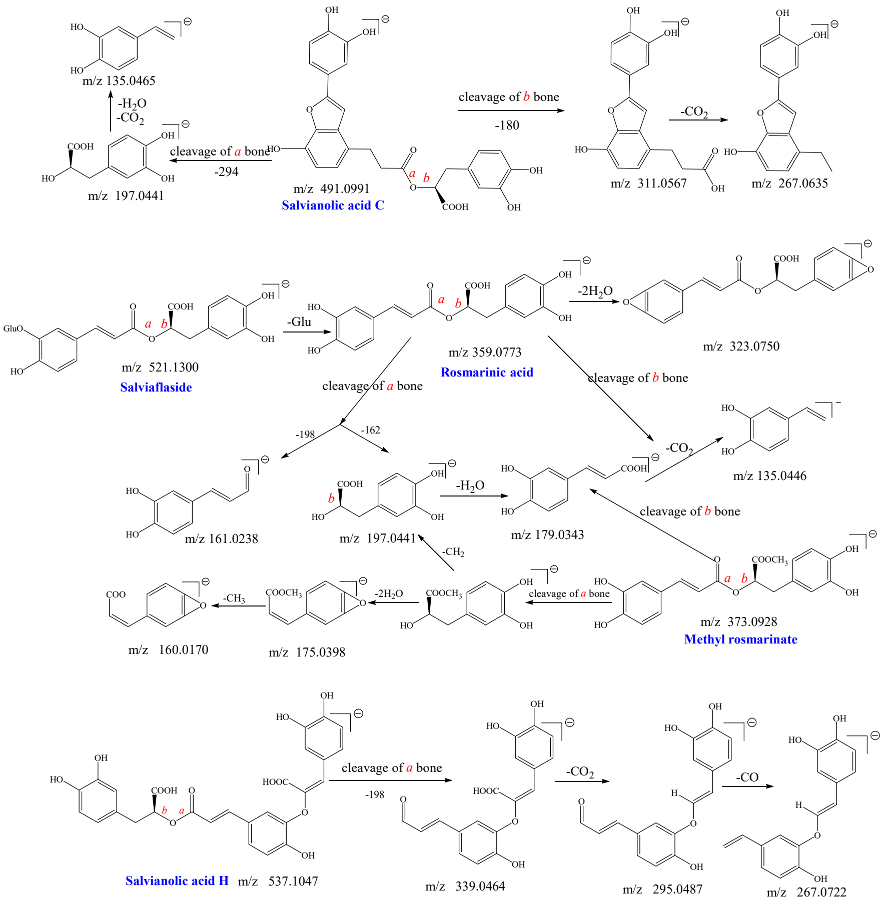

Figure S4 Possible mass fragmentation pathways of rosmarinic acid, salviafliside, methyl rosmarinate, salvianolic acid C and salvianolic acid H

Compound **17** given a precursor ion at m/z 491.0991 $[M-H]^-$. The fragment ions m/z 311.0567, 267.0635 and 135.0465 in the negative ion mode were the precursor ion (m/z 491.0991) after losing the tanshinol structural fragment, and the fragment ion

(m/z 311.0567) after losing CO_2 through secondary cleavage, and the precursor ion (m/z 491.0991) after losing the tanshinol structural fragment and then losing H_2O and CO_2, respectively. Therefore, the compound was identified as salvianolic acid C (**17**) (Liu et al., 2007), and its possible cracking pathway was inferred as shown in **Figure S3**. Compared with that of compound **9**, **12** and **14** (m/z 717.1470), the quasi-molecular ion peak (m/z 715.1301) of compound **13** was obtained after losing H_2. Therefore, compound **13** was the dehydrogenation product of compound **9**, **12** and **14** by comparing and the database GNPs platform, in-house traditional Chinese medicine (TCM) database.

The quasi molecular ion peaks of compounds **8**, **11** and **16** were m/z 521.1264, m/z 359.0742 and 373.0928, respectively. The main fragment ions were shown in **Table S2**. The pyrolysis characteristics of these three compounds suggested that compounds **8** and **11** had similar fragments of m/z 359.0742 and m/z 161.0238, compounds **16** and **11** had similar fragments of m/z 197.0423, m/z 179.0397 and m/z 123.0456, and compounds **8**, **11** and **16** had the same fragment m/z 135.0446. These indicated that the structures of the three compounds were similar. The cracking law of compound **11** was basically consistent with rosmarinic acid (Zeng et al., 2006), it was thus speculated that the compound was rosmarinic acid. The fragment ions in the ESI/MS^n mass spectrum of compound **8** revealed that the fragment ions (m/z 359.0740) were obtained by the loss of a dehydrated hexosyl (162) from the precursor ion (m/z 521.1264), indicating the existence of a glucose in compound **8**. It could be seen that compound **11** was produced by the cleavage of glycosidic bond of

compound **8**. According to the relevant reference (Lin et al., 2021), compound **8** was salviaflisside. The precursor ion (m/z 373.0928) of compound **16** was 14 different from that of compounds **11** (m/z 359.0742), which was a CH_2 structural unit. Therefore, compound **16** could be regarded as the methylation product of compound **11**. Combined with the mass spectrum characteristics of compound **16**, it could be inferred that the compound was methyl rosmarinate (Yang et al., 2019). The possible cleavage pathway of compounds **8**, **11** and **16** were shown in **Figure S3**.

The quasi molecular ion peaks of compound **2** was m/z 487.1458 $[M-H]^-$. Fragment ions m/z 163.0405 and m/z 145.0300 were obtained by losing a sophorae fragment in different fracture modes (Yang et al., 2019). Therefore, the compound was identified as *p*-hydroxycinnamic acid sophoroside. The precursor ion of compound **15** was m/z 343.0826 $[M-H]^-$, and the main fragment ions were m/z 191.0428, 181.0517, 161.0238, 135.0452 and 119.0515. The fragment (m/z 119.0515) was obtained by the loss of a tanshinol structural fragment (-198) and a neutral molecule (CO_2) in precursor ion, while m/z 191.0428 and 181.0517 were the fragments obtained from precursor ion by losing the tanshinol fragment in different ways, and fragment m/z 135.0452 was obtained from the loss of neutral molecules H_2O and CO_2 in fragment m/z 191.0428. This compound had the same characteristics with clinopodic acid A (Yang et al., 2019), this compound was thus inferred as clinopodic acid A (**Figure S4**).

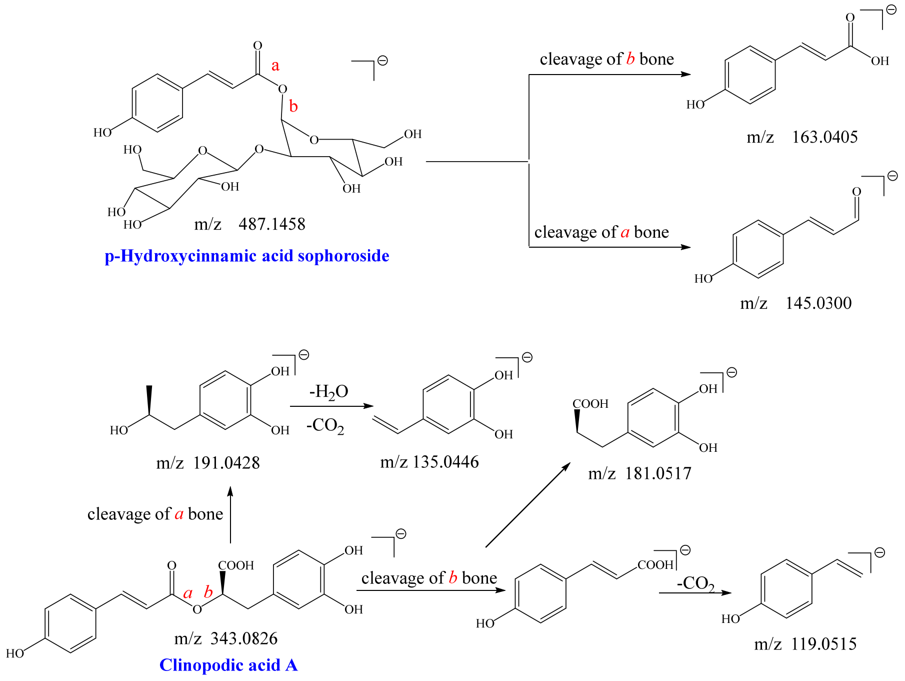

Figure S5 Possible mass fragmentation pathways of *p*-hydroxycinnamic acid sophoroside and clinopodic acid A

2. Flavonoids

Flavonoids are also the natural products widely distributed in nature, but these compounds have not been isolated from Radix *Salviae Miltiorrhizae*. A total of three flavonoids were identified from SMS meal, including compounds **7**, **18** and **19**, which were identified in SMS for the first time. The precursor ions of flavonoid mainly lost H_2O, CO, C_2H_2O, CO_2 and C_3O_2 to obtain corresponding fragment ions (Ding et al., 2011). In addition, according to RDA (Retro Diels-Alder reaction) theory, six membered ring compounds with double bonds could be decomposed into diene and diene friendly fragment ions under the action of ion sources (Ding et al., 2011).

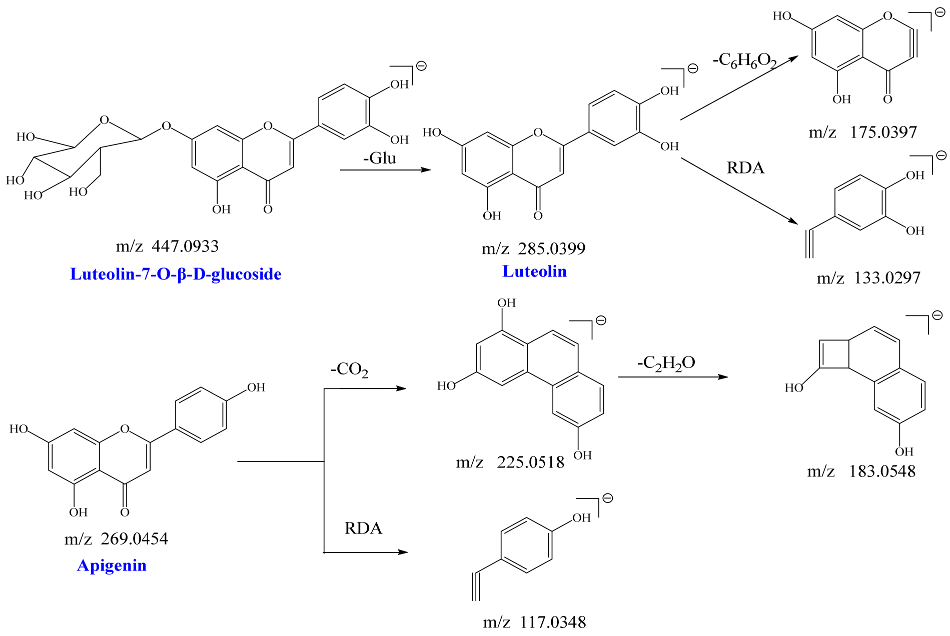

Fig. S6 Possible mass fragmentation pathways of apigenin, luteolin and luteolin-7-*O*-*β*-*D*-glucoside

The quasi molecular ion peak of compound **19** was m/z 269.0454 $[M-H]^-$, and the molecular formula was speculated to be $C_{15}H_{10}O_5$. The main fragment ions were m/z 225.0518, m/z 183.0548 and m/z 117.03, which correspond to $[M-H-CO_2]^-$, $[M-H-CO_2- C_2H_2O]^-$ and $[M-H-C_7H_4O_4]^-$, respectively. After matching with the database, it was speculated that the compound **19** was apigenin (Quirantes-Piné et al., 2013), and its possible mass spectrum cleavage pathway was shown in **Figure S5**. The precursor ion of compound **18** was m/z 285.0399 $[M-H]^-$, and the molecular formula was speculated to be $C_{15}H_{10}O_6$. The main fragment ions included m/z 175.0397, 149.0249 and 133.0297. Among them, m/z 175.0397 was the fragment obtained from the loss of $C_6H_6O_2$ of compound **18**, and m/z 133.0297 was the fragment obtained from the cracking of C ring of compound **18** by RDA. Therefore, compared with the literatures (Li et al., 2022; Quirantes-Piné et al., 2013), the

compound was determined as luteolin, and the secondary mass spectrum and possible cracking law were shown in **Figure S5**. Compared with compound **18**, the precursor ion of compound **7** was m/z 447.0932 $[M-H]^-$, and the main fragment was m/z 285.0379. It could be seen from the results that compounds **7** and **18** produced the same ion m/z 285.0379, which implied that they had the similar structure. Through the analysis of fragment ions in ESI/MS^n mass spectrometry of compound **7**, it was found that the fragment ion (m/z 285.0379) was obtained by the loss of a neutral fragment (dehydrated hexosyl, 162) from the precursor ion (m/z 447.0932), indicating that the compound **7** had a extra unit of glucose than compound **18**. Therefore, compound **7** was presumed to be luteolin-7-*O*-*β*-*D*-glucoside (Quirantes-Piné et al., 2013; Li et al., 2022).

3. Terpenoids

Terpenoids are also representative chemical components of Radix *Salviae Miltiorrhizae*. The terpenoids in Radix *Salviae Miltiorrhizae* are mainly diterpenes and contain a small amount of pentacyclic triterpenoids as well (Shan et al., 2021). Diterpenes are the characteristic components and the main active substances of Radix *Salviae Miltiorrhizae* (Shan et al., 2021). This study also identified three terpenoids (compounds **20-22**) from SMS meal.

The quasi molecular ion peak of compound **20** was m/z 487.3424 $[M-H]^-$, and the molecular composition was presumed to be $C_{30}H_{48}O_5$. Fragment ion peak m/z 469.3293 could be obtained after lost H_2O. Combined with literature and database search (Hu et al., 2020), the compound was identified as asiatic acid (**20**).

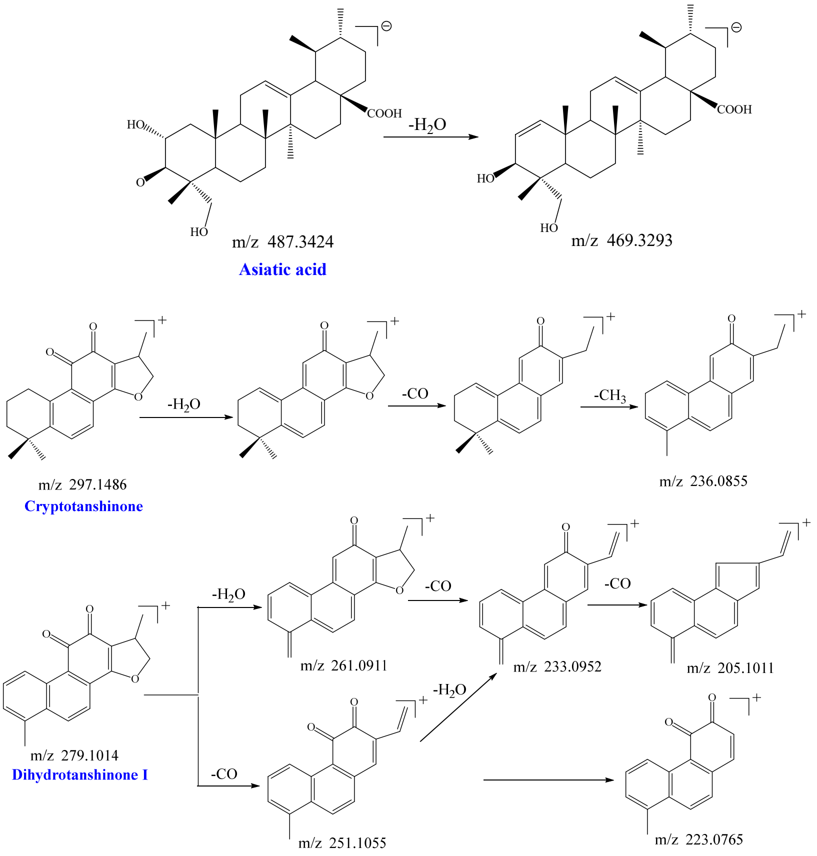

Figure S7 Possible mass fragmentation pathways of asiatic acid, dihydrotanshinone I and cryptotanshinone

In the positive model, the precursor ion of compound **21** was m/z 279.1014 $[M+H]^+$. the fragment ion m/z 251.1055, 261.0911, 205.1011 and 223.0765 were obtained by $[M+H- H_2O]^+$, $[M+H-CO]^+$, $[M+H-3CO]^+$ and $[M+H-H_2O-C_2H_4]^+$, respectively. It could be seen that this compound mainly broke down by losing neutral small molecules such as C_2H_4, H_2O and CO. These characteristics were consistent with dihydrotanshinone I (Liu et al., 2008), it was thus assumed that the compound was dihydrotanshinone I. The quasi molecular ion peak of compound **22** in positive mode was m/z 297.1486 $[M+H]^+$, and the molecular formula was $C_{19}H_{20}O_3$. Fragment ion m/z 236.0855 was formed by $[M+H-H_2O- CO -CH_3]^+$. It was found that the cracking law of this compound was consistent with that of cryptotanshinone (Chen, &

Xiong, 2016; Zhang, Hu et al., 2019), this compound was then determined to be cryptotanshinone, and its possible cracking pathway was shown in **Figure S6**.

4. Others compounds

In the negative mode, the precursor ion of compound **3** was m/z 387.1458 $[M-H]^-$, and the molecular composition was speculated to be $C_{18}H_{28}O_9$. Fragments m/z 207.0936 and 163.1129 were obtained by $[M-H-glucose-H_2O]^+$ and $[M-H-glucose-H_2O-CO_2]^+$. Combined with literature and database search (Šimko et al., 2016), the compound was identified as tuberonic acid glucoside (**3**).

The quasi molecular ion peak of compound **5** was m/z 521.1694 $[M-H]^-$, and the corresponding molecular formula was $C_{25}H_{30}O_{12}$. Fragment m/z 503.1556 was obtained by losing of neutral molecule H_2O from precursor ion, and fragment 325.0907 was obtained by the loss of iridoid units from precursor ion. Fragment m/z 265.0707 was obtained from precursor ion after the loss of H_2O, *p*-hydroxycinnamic acid and H_2O on glucose. Combined with literature and database search (Çaliş et al., 1993), the compound was identified as albidoside (**5**).

The precursor ion of compound **10** was m/z 535.1821 $[M-H]^-$, and the molecular composition was speculated to be $C_{25}H_{30}O_{12}$. The main fragment ions were m/z 373.1288, m/z 355.1191 and m/z 295.1067, which correspond to $[M-H-glucose]^-$, $[M-H-glucose -H_2O]^-$ and $[M-H-glucose-OCH_2]^-$, respectively. Fragment 179.0550 was the glucose unit. Combined with literature and database search (Liu et al., 2016), the compound was identified as pinoresinol glucoside (**10**).

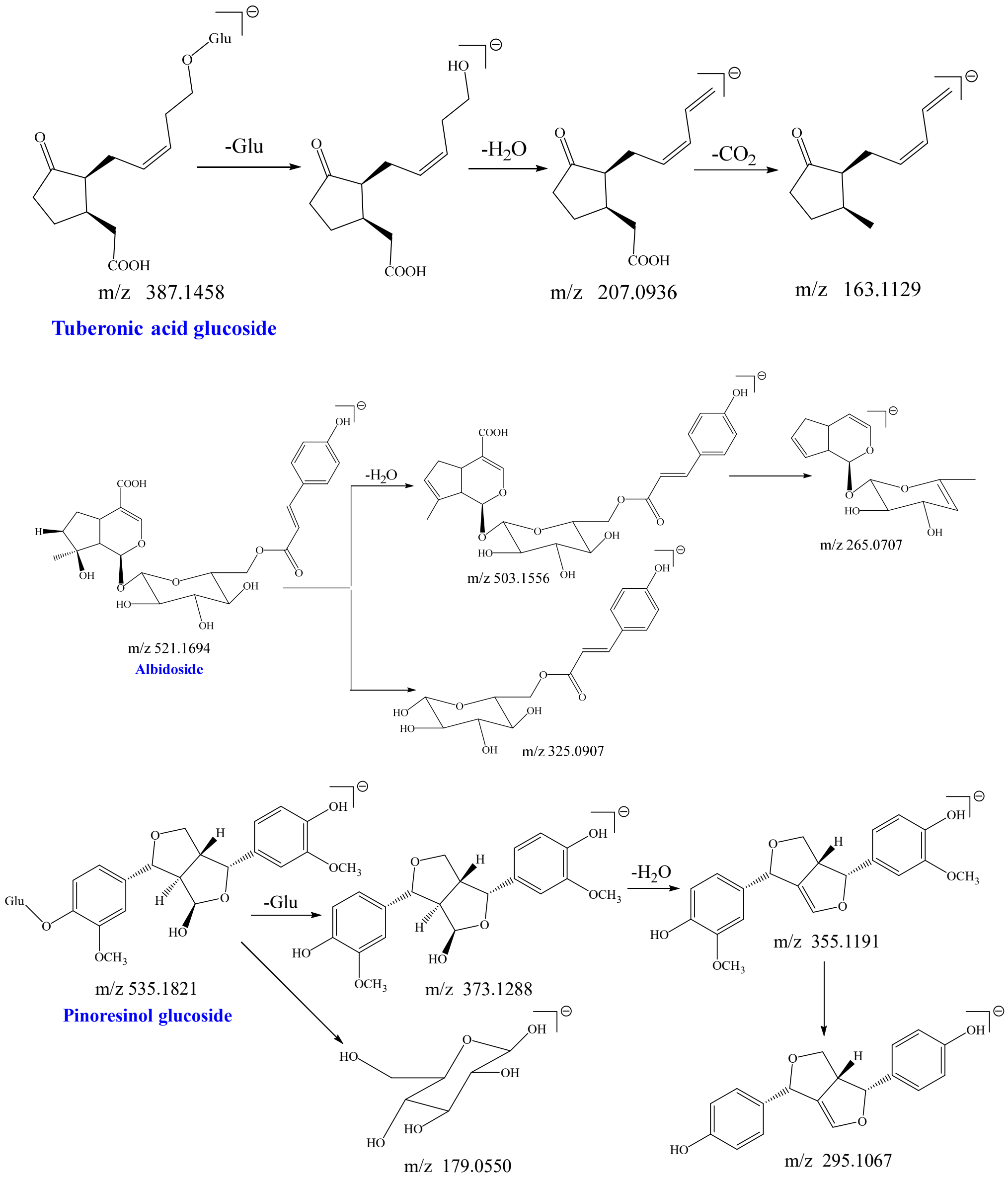

Figure S8 Possible mass fragmentation pathways of tuberonic acid glucoside, albidoside and pinoresinol glucoside

Text S2. The spectral data of the isolated compounds

Compound 1 (Ursolic acid**):** White powder. ESI-MS m/z: 457 $[M+H]^+$. ^{1}H-NMR (400 MHz, C_2D_5N) δ: 0.85 (3H, s), 0.97 (3H, s), 0.99 (3H, s, J = 6.7 Hz), 1.01 (3H, s), 1.03 (3H, d, J = 6.8 Hz), 1.23 (3H, d), 1.26 (3H, s), 2.64 (1H, d, J = 11.8 Hz, 18-H), 3.46 (1H, m, 3-H), 5.48 (1H, br s, 12-H); ^{13}C-NMR (100 MHz, C_5D_5N) δ: 38.2 (C-1), 27.9 (C-2), 77.9 (C-3), 39.3 (C-4), 55.6 (C-5), 18.6 (C-6), 33.4 (C-7), 42.3

(C-8), 47.8 (C-9), 37.2 (C-10), 23.7 (C-11), 125.4 (C-12), 139.1 (C-13), 42.3 (C-14), 28.6 (C-15), 24.7 (C-16), 47.8 (C-17), 53.3 (C-18), 33.4 (C-19), 38.9 (C-20), 30.9 (C-21), 37.1 (C-22), 28.6 (C-23), 16.4 (C-24), 15.5 (C-25), 17.2 (C-26), 23.7 (C-27), 179.7 (C-28), 17.3 (C-29), 21.2 (C-30).

Compound 2 (Tormentic acid**):** White powder. ESI-MS m/z: 489 $[M+H]^+$. ^{1}H-NMR (C_2D_5N, 400 MHz), δ: 1.02 (3H, s), 1.06 (3H, s), 1.09 (3H, s), 1.10 (3H, d, J = 6.7 Hz), 1.30 (3H, s), 1.44 (3H, s), 1.70 (3H, s), 5.57 (1H, br s, 12-H),3.09 (1H, s, 18-H), 4.13 (1H, m, 2-H), 3.39 (1H, d, J = 9.5 Hz, 3-H)。^{13}C-NMR (100 MHz, C_5D_5N) δ (C-1~30): δ: 48.0 (C-1), 68.4 (C-2), 83.6 (C-3), 39.6 (C-4), 55.7 (C-5), 18.9 (C-6), 33.3 (C-7), 40.2 (C-8), 48.0 (C-9), 39.3 (C-10), 23.9 (C-11), 127.7 (C-12), 139.8 (C-13), 42.1 (C-14), 29.1 (C-15), 26.1 (C-16), 47.7 (C-17), 54.4 (C-18), 72.4 (C-19), 41.9 (C-20), 26.8 (C-21), 38.2 (C-22), 18.0 (C-23), 29.7 (C-24), 17.0 (C-25), 17.7 (C-26), 24.5 (C-27), 180.5 (C-28), 26.7 (C-29), 16.7 (C-30).

Compound 3 (Salviaflaside**):** Light yellow powder, ESI-MS m/z: 523$[M+H]^+$. ^{1}H-NMR (CD_3OD, 400 MHz) δ: 7.63 (1H, d, J = 16.0 Hz, H-7), 6.40 (1H, d, J = 16.0 Hz, H-8), 7.56 (1H, br s, H-2), 6.89 (1H, br d, H-6), 7.21 (1H, d, H-5), 6.78 (1H, br s, H-2'), 6.72 (1H, d, J = 8.0 Hz, H-5'), 6.65 (1H, br d, J = 8.0 Hz, H-6'), 5.02 (1H, dd, J = 10.2, 1.5 Hz, H-8'), 3.04 (1H, dd, J = 14.0, 10.2 Hz, H-7'α), 3.10 (1H, br d, J = 14.0 Hz, H-7'β), 4.83 (1H, d, J = 7.4 Hz, H-1″), 3.33~3.54 (6H, m, H-2″~6″)。^{13}C-NMR (CD_3OD, 100 MHz) δ: (C-1~30): δ : 126.5 (C-1), 116.2 (C-2), 145.7 (C-3), 149.7 (C-4), 116.5 (C-5), 124.9 (C-6), 143.9 (C-7), 116.0 (C-8), 166.9 (C-9), 128.0 (C-1'), 116.5 (C-2'), 144.7 (C-3'), 143.9 (C-4'), 116.3 (C-5'), 120.4 (C-6'), 36.5 (C-7'), 73.4

(C-8'), 172.3 (C-9'), 102.7 (C-1''), 77.1 (C-2''), 73.5 (C-3''), 76.1 (C-4''), 70.1 (C-5''), 61.1 (C-9'').

Compound 4 (Rosmarinic acid): Light yellow oil. ESI-MS m/z: 361 $[M+H]^+$. ^{1}H-NMR (400 MHz, CD_3OD) δ: 6.78 (1H, d, *J* = 1.7 Hz, H-2), 6.74 (1H, d, *J* = 8.2 Hz, H-5), 6.65 (1H, dd, *J* = 8.2, 1.7 Hz, H-6), 3.10 (2H, m, H-7), 5.23 (1H, dd, *J* = 8.3, 4.3 Hz, H-8), 6.81 (1H, d, *J* = 8.4 Hz, H-5′), 7.08 (1H, d, *J* = 1.8 Hz, H-2′), 7.00 (1H, dd, *J* = 8.4, 1.8 Hz, H-6′), 7.59 (1H, d, *J* = 16.1 Hz, H-7′), 6.31 (1H, d, *J* = 16.1 Hz, H-8′); ^{13}C-NMR (100 MHz, CD_3OD) δ: 128.0 (C-1), 116.3 (C-2), 145.5 (C-3), 144.1 (C-4), 115.2 (C-5),120.4 (C-6), 36.7 (C-7), 73.5 (C-8), 126.4 (C-1′), 113.2 (C-2′), 145.0 (C-3′),148.4 (C-4′), 114.0 (C-5′), 121.9 (C-6′), 146.4 (C-7′), 115.1 (C-8′), 167.3 (C-9′).

Compound 5 (Cryptotanshinone): Orange red needle crystal (acetone), mp 180-182°C. ESI-MS m/z 297 $[M+H]^+$. ^{1}H NMR (400 MHz, $CDCl_3$) δ: 3.10 (2H, m, H-1),1.73 (2H, m, H-2),1.61 (2H, m, H-3), 7.62 (1H, d, *J* = 8.1 Hz, H-6), 7.45 (1H, d, *J* = 8.1 Hz, H-7), 3.52 (1H, m, H-15), 4.83 (1H, m, H-16α), 4.34 (1H, m, H-16β), 1.17 (3H, s, Me-17), 1.32 (3H, s, Me-18), 1.32 (3H, s, Me-19); ^{13}C-NMR (100 MHz, $CDCl_3$) δ: 29.2 (C-1), 18.7 (C-2), 37.1 (C-3), 34.2 (C-4), 152.0 (C-5), 132.2 (C-6), 122.1 (C-7), 128.0 (C-8), 126.3 (C-9), 143.2 (C-10), 184.0 (C-11), 175.0 (C-12), 118.0 (C-13), 170.2 (C-14), 34.1 (C-15), 80.9 (C-16), 18.1 (C-17), 31.2 (C-18), 31.1 (C-19).

Compound 6 (Dihydrotanshinone I): Red crystal, ESI-MS m/z: 279$[M+H]^+$. ^{1}H NMR (400 MHz, $CDCl_3$) δ: 9.25 (1H, d, *J* = 8.5 Hz, H-1) ,7.54 (1H, t, *J* = 8.5 Hz, H-2),

7.38 (1H, d, J = 8.5 Hz, H-3), 8.25 (1H, d, J = 8.5 Hz, H-6), 7.74 (1H, d, J = 8.5 Hz, H-7), 3.62 (1H, m, H-15), 4.94 (1H, t, J = 9.5 Hz, H-16α), 4.42 (1H, m, H-16β), 1.37 (3H, d, J = 6.8 Hz, Me-17), 2.70 (3H, s, Me-18); ^{13}C NMR (100 MHz, $CDCl_3$) δ: 124.7 (C-1), 130.3 (C-2), 128.6 (C-3), 134.7 (C-4), 134.5 (C-5), 131.7 (C-6), 122.0 (C-7), 130.5 (C-8), 125.6 (C-9), 135.2 (C-10), 184.0 (C-11), 175.6 (C-12), 118.5 (C-13), 170.7 (C-14), 34.5 (C-15), 81.3 (C-16), 18.5 (C-17), 19.7 (C-18).

Compound 7 (Apigenin): Yellow powder. ESI-MS m/z: 271$[M+H]^+$. ^{1}H-NMR (400 MHz, DMSO-d6) δ: 7.93 (2H, d, J = 8.8 Hz, H-2',6'), 6.94 (2H, d, J = 8.8 Hz, H-3', 5'), 6.80 (1H, s, H-3), 6.50 (1H, d, J = 2.0 Hz, H-8), 6.21 (1H, d, J = 2.0 Hz, H-6); ^{13}C-NMR (100 MHz, DMSO-d6) δ: 164.0 (C-2), 103.1 (C-3), 182.0 (C-4), 161.3 (C-5), 99.0 (C-6), 164.4 (C-7), 94.3 (C-8), 157.5 (C-9), 103.9 (C-10), 121.5 (C-1'), 128.7 (C-2', 6'), 116.0 (C-3', 5'), 161.7 (C-4').

Compound 8 (Luteolin):Yellow powder. ESI-MS m/z: 287$[M+H]^+$. ^{1}H-NMR (400 MHz, CD_3OD) δ: 7.43 (1H, d, J = 2.0 Hz, H-2'), 7.42 (1H, dd, J = 8.0, 2.0 Hz, H-6'), 6.91 (1H, d, J = 8.0 Hz, H-5'), 6.67 (1H, s, H-3), 6.45 (1H, d, J = 2.0 Hz, H-8), 6.20 (1H, d, J =2.0 Hz, H-6); ^{13}C-NMR (100 MHz, DMSO-d6) δ:164.1 (C-2), 103.0 (C-3), 181.7 (C-4), 157.4 (C-5), 99.0 (C-6), 164.4 (C-7), 94.1 (C-8), 161.7 (C-9), 103.8 (C-10), 121.7 (C-1'), 113.6 (C-2'), 145.9 (C-3'), 149.8 (C-4'), 116.2 (C-5'), 119.0 (C-6').

Compound 9 (Luteolin-7-O-β-D-glucoside): Yellow powder. ESI-HR-MS m/z: 449 $[M+H]^+$. ^{1}H-NMR (400 MHz, CD_3OD) δ: 6.90 (1H, s, H-3), 6.82 (1H, d, J = 2.0 Hz, H-6), 6.99 (1H, d, J = 2.0 Hz, H-8),7.90 (1H, d, J = 2.2 Hz, H-2'), 7.26 (1H, d, J = 8.4

Hz, H-5'), 7.51 (1H, d, *J* =8.4, 2.2 Hz, H-6'), 5.9 (1H, d, *J* = 7.6 Hz, H-1"), 4.36 (1H, t, *J* = 8.6 Hz, H-2"), 4.41 (1H, t, *J* = 8.6 Hz, H-3"), 4.33 (1H, t, *J* = 8.6 Hz, H-4"), 4.18 (1H, t, *J* = 8.6 Hz, H-5"), 4.57 (1H, m, H-6"a), 4.39 (1H, m, H-6" b). ^{13}C-NMR (CD_3OD, 125 MHz) δ: 165.3 (C-2), 104.1 (C-3), 185.6 (C-4), 162.5 (C-5), 100.5 (C-6), 163.8 (C-7), 95.1 (C-8), 157.5 (C-9), 106.5 (C-10), 122.5 (C-1'), 114.6 (C-2'), 147.7 (C-3'), 151.6 (C-4'), 116.6 (C-5'), 119.5 (C-6'), 101.6 (C-1"), 74.6 (C-2"), 78.3 (C-3"),71.0 (C-4"), 79.2 (C-5"), 62.2 (C-6").

Compound 10 (Tanshinol): Translucent colloid. ESI-HR-MS m/z: 199 $[M+H]^+$. ^{1}H-NMR (400 MHz, DMSO-d6) δ: 6.66 (1H, d, *J* = 2.1 Hz, H-2), 6.63 (1H, d, *J* = 8.2 Hz, H-6), 6.47 (1H, m, H-5), 4.08 (1H, m, H-8), 2.80 (1H, m, H-7), 2.63 (1H, m, H-7'); ^{13}C-NMR (75 MHz, DMSO-d6) δ: 120.4 (C-1), 117.2 (C-2), 143.7 (C-3), 144.9 (C-4), 115.5 (C-5), 129.1 (C-6), 39.9 (C-7), 71.8 (C-8), 175.6 (C-9).

Compound 11 (Salvianolic acid B**):** White powder, ESI-MS m/z: 719$[M+H]^+$. ^{1}H-NMR (CD_3OD, 400 MHz) δ: 6.85 (1H, d, *J* = 8.4 Hz, H-5), 7.19 (1H, d, *J* = 8.4 Hz, H-6), 7.55 (1H, d, *J* = 16.0 Hz, H-7), 6.24 (1H, d, *J* = 16.0 Hz, H-8), 6.54 (1H, d, *J* = 2.1 Hz, H-2′), 6.58 (1H, d, *J* = 2.1 Hz, H-5′), 6.35 (1H, m, H-6′), 2.85 (1H, m, H-7′a), 3.10 (1H, m, H-7′b), 5.21 (1H, m, H-8′), 6.81 (1H, d, *J* = 2.1 Hz, H-2″), 6.73 (1H, d, *J* = 8.2 Hz, H-5″), 6.69 (1H, m, H-6″), 5.63 (1H, d, *J* =5.4 Hz, H-7″), 4.39 (1H, d, *J* =5.4 Hz, H-8″), 6.78 (1H, d, *J* =8.2 Hz, H-5‴), 6.66 (1H, m, H-6‴), 3.05 (2H, m, H-7‴), 5.21 (1H, m, H-8‴). ^{13}C-NMR (CD_3OD, 100 MHz) δ: 124.8 (C-1), 126.5 (C-2), 146.9 (C-3), 149.2 (C-4), 118.7 (C-5), 122.1 (C-6), 143.9 (C-7), 117.8 (C-8), 168.5 (C-9), 129.3 (C-1'), 116.8 (C-2'), 146.7 (C-3′), 146.3 (C-4′), 116.9 (C-5′),

122.5 (C-6′), 38.2 (C-7′), 75.0 (C-8′), 173.8 (C-9′), 133.9 (C-1″), 113.8 (C-2″), 146.2 (C-3″), 145.6 (C-4″), 116.6 (C-5″),117.7 (C-6″), 88.5 (C-7″), 58.1 (C-8″), 172.7 (C-9″), 129.6 (C-1‴), 116.7 (C-2‴), 145.4 (C-3‴), 145.2 (C-4‴), 118.7 (C-5‴), 122.7 (C-6‴), 37.7 (C-7‴), 76.0 (C-8‴), 172.6 (C-9‴).

References

Ammar, R.B., Bhouri, W., Sghaier, M.B., Boubaker, J., Skandrani, I., Neffat, A., Bouhlel, I., Kilani, S., Mariotte, A.M., Leila, C.G., Dijoux-Franca, M.G., Ghedira, K. (2009). Antioxidant and free radical-scavenging properties of three flavonoids isolated from the leaves of Rhamnus alaternus L. (Rhamnaceae): A structure-activity relationship study. Food Chem., 116, 258-264. https://doi.org/10.1016/j.foodchem.2009.02.043

Chen, Q. H., & Xing, L. (2016). Fragmentation pathways of cryptotanshinone revealed by Ion Trap Mass Spectrometry in positive mod. Chin. Med. J. Res. Prac., 30(1), 9-12.

https://doi.org/10.13728/j.1673-6427.2016.01.004

Ding, J. H., Wang, X. X., Zhang, H., Pan, S. S., Luo, M. B., Li, J. Q., & Chen, H. W. (2011). Extrative electrospray ionization Tandem Mass Spectrometry of apigenin. Chem. J. Chinese U., 32(8), 1714-1719.

Gan, G. S., Cao, C., Gan, Z. Y., Zuo, Q. (2018). Effect of oil refining process and decolorization conditions on Soybean oil quality. J. Anhui Sci. Technol. Univ., 32, 50-55.

https://doi.org/10.19608/j.cnki.1673-8772.2017.0539

Gao, L., Hu, B. R., Qi, Y. L., Zhu, Y. N. (2009). Grape seed oil: organic solvent extraction with the assistance of cellulase or ultrasonic and evaluation of physico-chemical properties. Food Science, 30, 81-83.

Fu, R., Zhang, Y.T., Guo, Y. R., Liu, F. X., Chen, F. (2014). Determination of phenolic contents and antioxidant activities of extracts of *Jatropha curcas* L. seed shell, a by-product, a new source of natural antioxidant. Ind. Crop. Prod., 58, 265-270.

https://doi.org/10.1016/j.indcrop.2014.04.031

Gan, G. S., Cao, C., Gan, Z. Y., Zuo, Q. (2018). Effect of oil refining process and decolorization conditions on Soybean oil quality. J. Anhui Sci. Technol. Univ., 32, 50-55. https://doi.org/10.19608/j.cnki.1673-8772.2017.0539

Gao, L., Hu, B. R., Qi, Y. L., Zhu, Y. N. (2009). Grape seed oil: organic solvent extraction with the assistance of cellulase or ultrasonic and evaluation of physico-chemical properties. Food Science, 30, 81-83.

Hui, R.H., Hou, D.Y., Li, X. C., Liu, X. Y., Han, Y. (2006). Preparation of corn oil and determination of fatty acids in corn oil. Food Science, 27, 418-450.

Li, Y., Li, Y. J., Zhang, G. Q., Li, H. Y., & Jing, M. (2022). Analysis of chemical constituents in *Biebersteinia heterstemon* Maxim by HPLC-Q-TOF-MS/MS. Chin. J. Ethnomedi. Ethnopharm., 31(9), 45-52.

Liu, Y. J., Chen, Y. H., Yang, X. S., Ren, G. X. (2020). Research progress on nutrition and functional components of millet. Cereals & Oils, 33, 1-3.

Tai, Z.G., Chen, A.Y., Qin, B.D., Cai, L., Xu, Y.Q. (2014). Chemical constituents and antioxidant activity of the *Musa basjoo* flower. Eur. Food Res. Technol, 239, 501-508.

Taghvaei, M., Jafari, S. M., Assadpoor, E., Nowrouzieh, S., & Alishah, O. (2014). Optimization of microwave-assisted extraction of cottonseed oil and evaluation of its oxidative stability and physicochemical properties. Food Chem., 160, 90-97.

Yu, M., Zhou, Y. T., Ma, J. H., Shi, T. Y., Lu, M., Fu, X., Zhang, L. C. (2019). Study on the physicochemical properties and antioxidant activity of two varieties cold-pressed peanut oil. Food Research and Development, 40, 38-43.

Zhang, Y. N., Guo, L., Su, Y., Li, X. D. (2019). The Process of ultrasound and freeze-microwave assisted aqueous enzymatic extraction of sesame oil. Food Research and Development, 40, 102-109.

Zhang, Y. Y., Chen, F. X., Gu B., Liang, A. Y. (2015). Physicochemical properties and chemical composition of high oleic sunflower oil. Cereals and Oils Processing, 6, 34-36.

Yu, M., Zhou, Y. T., Ma, J. H., Shi, T. Y., Lu, M., Fu, X., & Zhang, L. C. (2019). Study on the physicochemical properties and antioxidant activity of two varieties cold-pressed peanut oil. Food Research and Development, 40, 38-43.
